# Cerebellar development after preterm birth

**DOI:** 10.3389/fcell.2022.1068288

**Published:** 2022-11-29

**Authors:** Igor Y. Iskusnykh, Victor V. Chizhikov

**Affiliations:** Department of Anatomy and Neurobiology, University of Tennessee Health Science Center, Memphis, TN, United States

**Keywords:** preterm birth, cerebellum, neurogenesis, granule cells, Purkinje cells, glia, hemorrhage, inflammation

## Abstract

Preterm birth and its complications and the associated adverse factors, including brain hemorrhage, inflammation, and the side effects of medical treatments, are the leading causes of neurodevelopmental disability. Growing evidence suggests that preterm birth affects the cerebellum, which is the brain region involved in motor coordination, cognition, learning, memory, and social communication. The cerebellum is particularly vulnerable to the adverse effects of preterm birth because key cerebellar developmental processes, including the proliferation of neural progenitors, and differentiation and migration of neurons, occur in the third trimester of a human pregnancy. This review discusses the negative impacts of preterm birth and its associated factors on cerebellar development, focusing on the cellular and molecular mechanisms that mediate cerebellar pathology. A better understanding of the cerebellar developmental mechanisms affected by preterm birth is necessary for developing novel treatment and neuroprotective strategies to ameliorate the cognitive, behavioral, and motor deficits experienced by preterm subjects.

## Introduction

Preterm birth is a significant medical condition that is the leading cause of neonatal mortality and infant morbidity worldwide (Tosto et al., 2020). The World Health Organization (WHO) defines preterm birth as birth before 37 weeks of a typical 40-week human pregnancy (Samuel et al., 2019). The rate of preterm birth varies from 5% to 18% of human pregnancies, depending on the geographic region. Low-income areas have the highest rate of preterm birth, and its incidence is growing worldwide (Tosto et al., 2020).

Preterm birth is associated with a wide range of long-lasting adverse health outcomes, particularly neuropsychological deficits that include impairment of cognitive, behavioral, language, socialization, or motor-coordination functions. It is estimated that one or more of such neuropsychological problems develop in 25–50% of preterm infants (Volpe, 2009, 2021; Lammertink et al., 2020).

Due to their high social and medical importance, the origin of neurological problems associated with preterm birth has been extensively investigated in clinical and preclinical settings. At least some such deficits result from disruption of the cerebellum, the brain region involved in motor coordination, cognition, learning, memory, and social communication (Salman and Tsai, 2016; Sathyanesan et al., 2019; Guell and Schmahmann, 2020; van der Heijden et al., 2021). Numerous imaging studies report reduced cerebellar volume and/or altered cerebellar shape in preterm infants in both short and long-term observations. Cerebellar pathology in preterm infants involves both white and grey matter (Allin et al., 2001; Bouyssi-Kobar et al., 2016; Kim et al., 2016; Tam, 2018; Wu et al., 2020). Analysis of postmortem cerebellar samples from preterm subjects revealed specific deficits in several cerebellar cell types, further supporting the disruption of the cerebellar developmental program by preterm birth (Haldipur et al., 2011). It needs to be noted, however, that preterm infants frequently experience the adverse effects of a wide range of additional insults and negative factors, including but not limited to hemorrhage, infections/inflammation, and the side effects of medical treatments. Although these factors are not unique to preterm babies, their frequency is much higher in preterm than in term infants (Volpe, 2009).

In light of the aforementioned confounding factors that accompany preterm birth and the limited availability of human cerebellar tissue samples, it was difficult to identify the origin and developmental mechanisms of cerebellar pathologies that were directly attributable to preterm birth based solely on human patient analysis. In recent years, however, the problem has been alleviated by the use of animal models, especially large animal models, whose cerebellar developmental programs are particularly similar to those in humans. In this review, we will first briefly describe cerebellar anatomy and development. Then, we will discuss experimental approaches and models used to analyze the effects of preterm birth on the cerebellum. Finally, we will describe specific developmental mechanisms disrupted by preterm birth and some adverse factors commonly associated with preterm birth.

## Cerebellar neuroanatomy

Neuroanatomy and development of the cerebellum have been most studied in the mouse but appear to be sufficiently conserved across mammalian species (Chizhikov and Millen, 2003). Thus, we will first summarize cerebellar anatomy using the mouse as an example and then highlight some notable features specific to the cerebellum of humans.

**FIGURE 1:**
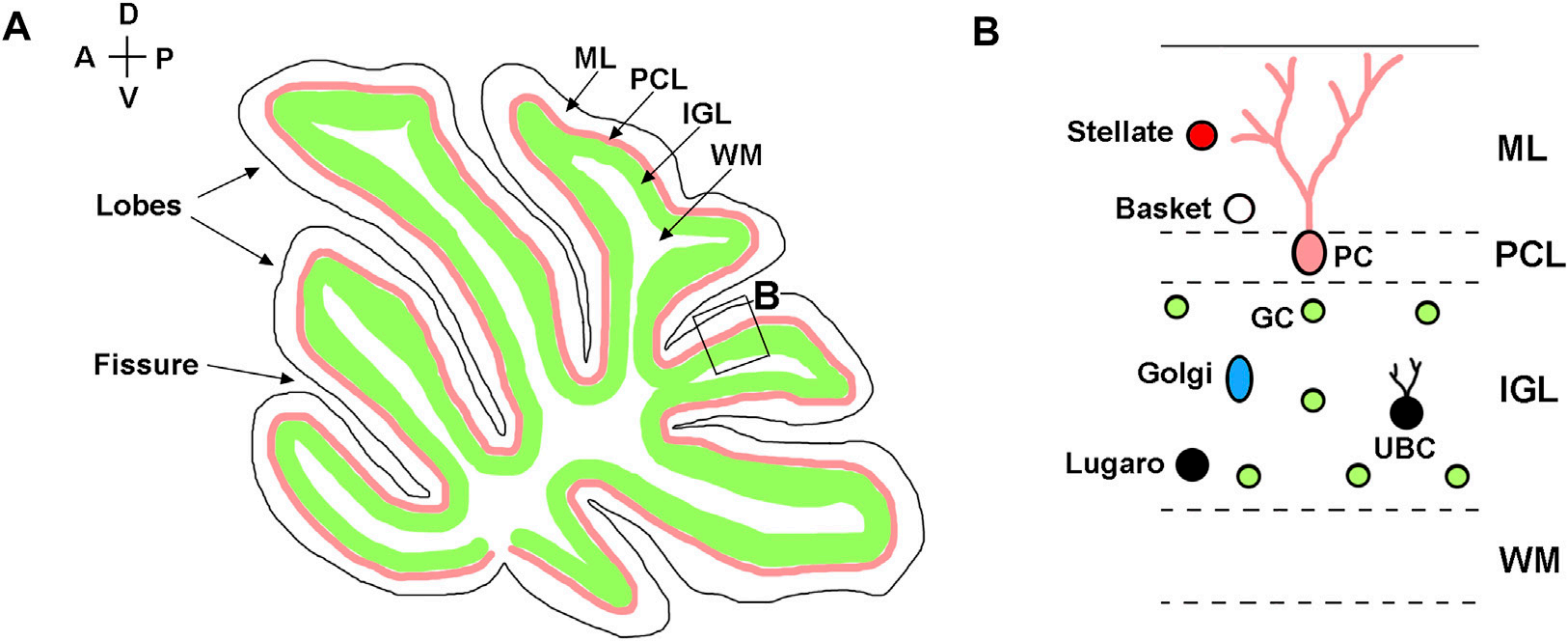
Neuroanatomy of adult cerebellum. **(A)** Diagram of a sagittal section of the adult mouse cerebellum, showing layered structure of the cerebellar cortex, which is subdivided by parallel fissures into distinct lobes. **(B)** Higher magnification of the region boxed in panel **(A)**. Molecular layer—ML; Purkinje cell layer—PCL; internal granule layer—IGL; white matter—WM; Purkinje cells—PC; granule cells—GC; Basket cells—Basket; Stellate cells—Stellate; Lugaro cells—Lugaro; Golgi cells—Golgi; Unipolar brush cells—UBC. The anterior-posterior (A–P) and dorsal-ventral (D–V) axes are shown in the top left corner.

Anatomically, mouse cerebellum is divided into two lateral hemispheres and a vermis that is located between the hemispheres. Both vermis and hemispheres are subdivided by fissures into distinct folia, forming species-specific foliation patterns (Figure 1A) (Chizhikov and Millen, 2020). The cerebellar cortex is comprised of three layers: the outermost molecular layer, the intermediately located Purkinje cell layer, and the innermost internal granule cell layer (IGL). The Purkinje cell layer contains the bodies of Purkinje neurons that provide the sole output of the cerebellar cortex. The molecular layer contains the dendrites of Purkinje cells and inhibitory basket and stellate neurons that innervate Purkinje cells. The IGL is primarily comprised of granule neurons (granule cells) that send excitatory signals to Purkinje cells and also contains unipolar brush cells, Lugaro cells, and Golgi cells (Figure 1B). A centrally located white matter contains cerebellar nuclei composed of excitatory and inhibitory neurons (Butts et al., 2014; Chizhikov and Millen, 2020; Marzban et al., 2014).

Although many of the aforementioned aspects of mouse cerebellar neuroanatomy also apply to the human cerebellum, certain species-specific cerebellar features exist in humans. For example, the human cerebellum has a surface area that is 750-fold larger than that of the mouse cerebellum. In addition, the human cerebellum exhibits greater folial complexity than the mouse cerebellum, and the human cerebellar hemispheres are enlarged relative to the vermis. Finally, the cerebellum contains approximately 80% of all neurons in the human brain, while it contains only 60% of the neurons in the mouse brain (Haldipur et al., 2022).

## Cerebellar development

Cerebellar development has been most extensively studied in mice. Thus, we will summarize cerebellar development, primarily based on mouse studies. Then, we will discuss developmental features unique to the human cerebellum.

**FIGURE 2:**
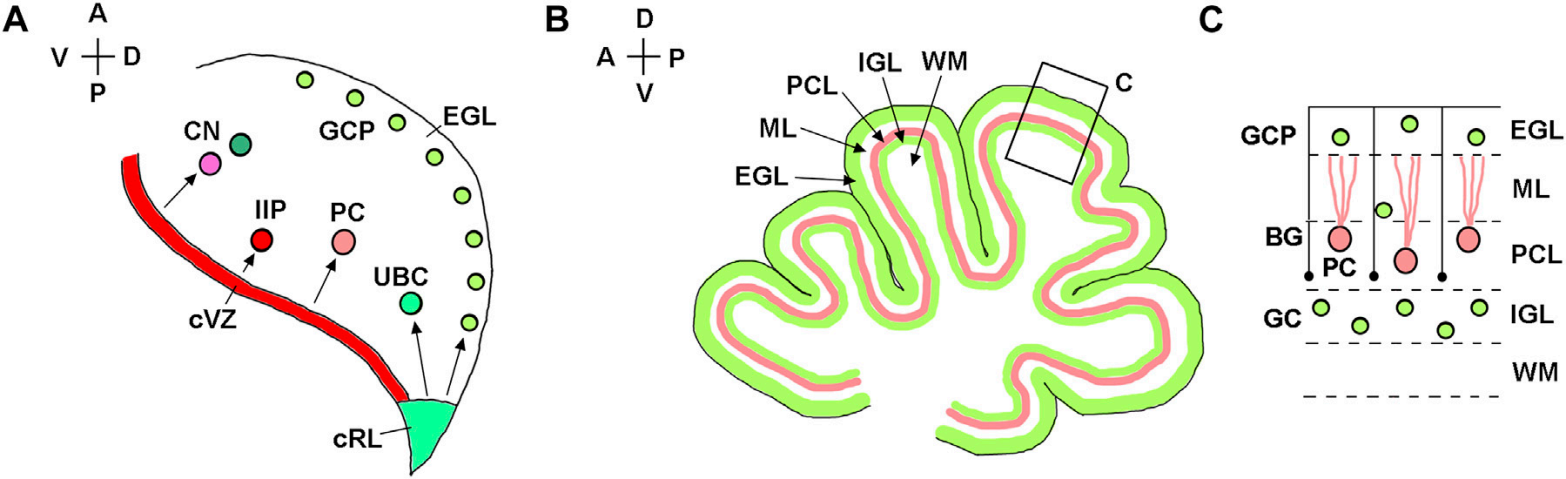
Developmental origin of major types of cerebellar neurons. **(A)** Diagram of a sagittal section of the mouse embryonic cerebellar anlage (e10.5-e18) showing that the cerebellar rhombic lip (cRL) gives rise to excitatory cerebellar cells, namely granule cell precursors (GCP), unipolar brush cells (UBC), and excitatory neurons of the cerebellar nuclei (CN). The cerebellar ventricular zone (cVZ) gives rise to inhibitory cells, such as Purkinje cells (PC), inhibitory interneuron progenitors (IIP) and inhibitory neurons of CN. Tangentially migrating GCPs form the secondary germinal zone—the external granule cell layer (EGL). **(B)** Diagram of a midsagittal section of the neonatal mouse cerebellum, showing layered organization of the cerebellar cortex. **(C)** Higher magnification of the region boxed in panel **(B)**. After transient amplification in the EGL, GCPs differentiate into granule cells (GC) and migrate radially along Bergmann glial fibers (shown as black lines) from the EGL to the IGL. External granule cell layer—EGL; Molecular layer—ML; Purkinje cell layer—PCL; internal granule layer—IGL; white matter—WM. The anterior-posterior (A–P) and dorsal-ventral (D–V) axes are shown in the top left corner of panels **(A)** and **(B)**.

In both mice and humans, during embryonic development, the cerebellum originates from rhombomere 1, the most anterior segment of the hindbrain (Sillitoe and Joyner, 2007; Chizhikov et al., 2021). The isthmic organizer located at the mesencephalon/rhombomere 1 boundary directs the formation of cerebellar territory through the fibroblast growth factor 8 (Fgf8) signaling pathway (Joyner et al., 2000; Nakamura et al., 2008; Yaguchi et al., 2009; Tong et al., 2015). In both spicies, establishment of the cerebellar territory is followed by the formation of two germinal zones with distinct developmental properties, namely the cerebellar ventricular zone and the cerebellar rhombic lip. The cerebellar ventricular zone is defined by expression of the pancreas associated transcription factor 1a (Ptf1a) and generates inhibitory cerebellar neurons (Figure 2A) (Hoshino et al., 2005; Pascual et al., 2007; Millen et al., 2014; Yamada et al., 2014; Haldipur et al., 2022; Khouri-Farah et al., 2022). The cerebellar rhombic lip is defined by the expression of atonal bHLH transcription factor 1 (Atoh1) and generates excitatory cerebellar neurons (Figure 2A) (Machold and Fishell, 2005; Wang et al., 2005; Englund et al., 2006; Fink et al., 2006; Yeung et al., 2014; Yeung et al., 2016; McDonough et al., 2020).

**FIGURE 3:**
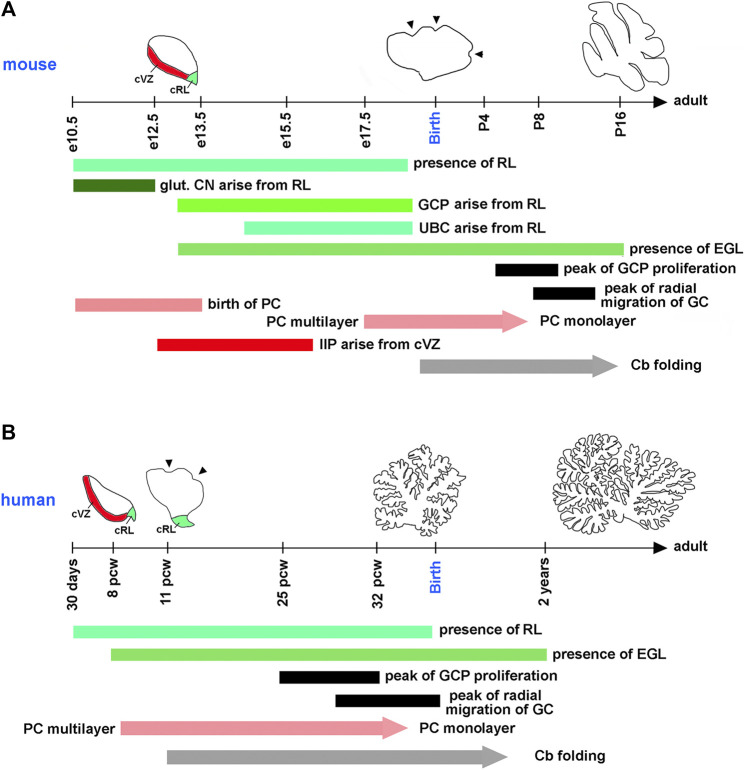
Timing of key cerebellar developmental events in the mouse and human. Horizontal axes show the mouse **(A)** and human **(B)** developmental timelines. On the mouse timeline, e and P mean embryonic and postnatal days, respectively. On the human timeline, pcw means postconceptual weeks. Diagrams showing the extent of cerebellar folding at selected developmental stages are shown above the timeline for each species. cRL and cVZ are shown at appropriate stages. Arrowheads point to fissures that begin forming on the outer cerebellar surface at species-specific developmental stages. Horizontal bars indicate the duration of distinct developmental processes in each species. Since the refinement of Purkinje cells into the monolayer and the development of cerebellar foliation (Cb folding) are gradual processes, they are shown as arrows. glut. CN—glutamatergic neurons of cerebellar nuclei. All other abbreviations are defined in legends for Figures 1, 2. Compared to the mouse, human embryonic cerebellar development is highly protracted. Many key cerebellar developmental events that occur in the mouse after birth, in humans are initiated or even completed *in utero*.

A typical mouse pregnancy lasts 19 days. Excitatory neurons that populate adult mouse cerebellar nuclei arise from the rhombic lip between embryonic days (e) 10.5 and 12.5. Granule cell precursors (GCPs) migrate from the rhombic lip between e13 and e18.5, when the rhombic lip regresses in the mouse (Machold and Fishell, 2005; Wang et al., 2005) (Figure 3A). After exiting the rhombic lip, GCPs populate the outer surface of the cerebellar anlage, forming a secondary germinal zone called the external granule cell layer (EGL) (Figure 2), where GCPs proliferate extensively before beginning their differentiation and radial migration along the Bergmann glial fibers (Basson and Wingate, 2013; Leung and Li, 2018; Consalez et al., 2020; Lowenstein et al., 2022). In the mouse, radial migration of granule cells begins around the time of birth (postnatal day 0, P0), when granule cells pass the Purkinje cell layer and settle in the IGL (Figures 2B,C). Mouse granule cell migration concludes by P16, when all GCPs have migrated from the EGL to IGL, leading to the disappearance of the EGL. Since amplification of GCPs is a significant contributor to the cerebellar foliation, the mature foliation pattern is established by P16 as well (Leto et al., 2016). Unipolar brush cells migrate from the rhombic lip into the white matter between e14.5 and e18.5 (Englund et al., 2006) (Figure 3A).

In the mouse, postmitotic Purkinje cells arise from the cerebellar ventricular zone between e10.5 and e13.5. By e17.5, these cells have migrated radially to form a cell cluster below the EGL that becomes refined into the monolayer several days following birth (Chizhikov and Millen, 2020). Murine progenitors of inhibitory interneurons exit the cerebellar ventricular zone between e12.5 and e16 and transiently amplify in the embryonic and neonatal cerebellar white matter, most extensively during P2-P8, before differentiating into mature Golgi, basket, and stellate cell types (Fleming et al., 2013; Leto et al., 2016; Li et al., 2022) (Figures 2A, 3A).

Although many aspects of cerebellar neurogenesis are conserved across mammalian species, notable differences exist between the cerebellum of humans and mice. These include significant differences in the cerebellar developmental trajectory, with mouse cerebellar development appearing “delayed” relative to that of human (Figures 3A,B). For example, many key developmental processes in the mouse cerebellum occur either postnatally, such as the peak of GCP proliferation, the refinement of Purkinje cells in the monolayer, and the onset of cerebellar foliation, or around the time of birth, such as the beginning of the migration of granule cells from the EGL to the IGL. In contrast, all these processes are either fully underway (extensive proliferation of GCPs, radial migration of granule cells, and folding of the cerebellar cortex) or conclude (refinement of Purkinje cells in the monolayer) in the third trimester of a typical 40-week-long human pregnancy (ten Donkelaar et al., 2003; ten Donkelaar and Lammens, 2009; Volpe, 2009; Haldipur et al., 2022) (Figures 3A,B). Recent studies have also described specific populations of progenitor cells in the rhombic lip and cerebellar subventricular zone that are present in the human cerebellum but absent in the mouse (Haldipur et al., 2019; Haldipur et al., 2022; Smith et al., 2022).

Although each cerebellar cell type employs a unique molecular program, it is essential to note that the development of different classes of cerebellar neurons depends on each other in both humans and mice. For example, Purkinje cells secrete the sonic hedgehog (Shh) protein that non-autonomously promotes proliferation of both GCPs in the EGL and inhibitory interneuron progenitors in the white matter (Dahmane and Ruiz i Altaba, 1999; Wallace, 1999; Wechsler-Reya and Scott, 1999; Fleming et al., 2013; Leto et al., 2016; Haldipur et al., 2022; Li et al., 2022). Thus, Purkinje cells act as a bi-directional signaling center that “scales” the populations of functionally connected cerebellar neurons. On the other hand, GCPs migrating tangentially over the cerebellar surface are known to attract Purkinje cells, and the survival of Purkinje cells is critically dependent on their proper innervation by molecular layer interneurons (Jensen et al., 2002; Edamakanti et al., 2018). Thus, while preterm birth and its associated factors may initially affect only one or few types of cerebellar cells, their effects may eventually become more global because of the interdependence of cerebellar neurons.

## Experimental approaches to study the preterm cerebellum

Human brain imaging studies were instrumental in revealing the adverse effect of preterm birth on cerebellar development, and analysis of postmortem human cerebellar tissue has begun identifying candidate cerebellar populations and the developmental mechanisms affected by preterm birth. However, interpretation of the results of human cerebellar postmortem tissue studies is complicated by numerous confounding factors, including hemorrhage, inflammation, and medical treatments, thereby necessitating the use of more controlled experimental animal models.

Mouse models provided important insights into the mechanisms of neural pathology caused by adverse factors associated with preterm birth. However, the mouse is not best suited to study preterm cerebellar neurogenesis because of the considerable natural variation of gestational length between dams, the difficulties in accurately assessing gestational length, extremely poor survival of preterm pups, and the discussed above differences in the cerebellar developmental trajectories in humans and mice (McCarthy et al., 2018; Haldipur et al., 2022). Thus, mouse studies of prematurity-related mechanisms must be interpreted with caution and, ideally, should be complemented by more translational large animal models.

While non-human primates are an excellent model for studying preterm birth (Rees et al., 2009; Barron and Kim, 2020), the extent of their use is limited by ethical considerations, their high cost, and the small number of offspring (typically one or two) resulting from each pregnancy. Other models that have been successfully used for preclinical studies relevant to humans include rabbits, guinea pigs, lambs, and pigs (Yawno et al., 2007; Sveinsdóttir et al., 2017; Iskusnykh et al., 2018; Shaw et al., 2018; Romantsik et al., 2019; Yawno et al., 2019; Iskusnykh et al., 2021; Vanden Hole et al., 2021). The pig is a particularly advantageous model for studying preterm birth. Pigs produce large litters of 7–15 fetuses per pregnancy, and preterm piglets are compatible with neonatal intensive care unit protocols and equipment used to care for preterm babies (Sangild et al., 2013; Buddington et al., 2018; Buddington et al., 2021). Importantly, analysis of the cerebellum of newborn pigs revealed a complex foliation pattern, a monolayer of Purkinje cells, and a well-developed IGL, indicating that similar to humans, in the pig, refinement of Purkinje cells in the monolayer, the onset of cerebellar foliation, the extensive proliferation of GCPs, and the radial migration of granule cells to a large extent occur *in utero* (Choudhri et al., 2014; Iskusnykh et al., 2018; Iskusnykh et al., 2021).

## The impact of preterm birth on different cerebellar cell types

Below we discuss what is known regarding the effect of preterm birth on specific cerebellar cell types and the molecular mechanisms mediating cerebellar cell-type specific pathology.

### Granule cells

As granule cells, by far, are the most numerous neurons in the cerebellum, and most cases of human cerebellar hypoplasia are characterized by a reduced number of granule cells (Hoshino et al., 2005; Aldinger et al., 2009; Lee and Gleeson, 2011; Haldipur et al., 2021), reduced cerebellar volume in preterm subjects is expected to involve granule cell abnormality. Indeed, a decreased number of granule cells in the IGL was observed in the cerebellum of preterm pigs, and lobe-specific reduction of the width of the IGL was described in preterm guinea pigs (Iskusnykh et al., 2018; Shaw et al., 2018). Several processes are critical to achieving an appropriate number of granule cells in the IGL, including the proper proliferation of GCPs in the EGL, normal differentiation of granule cells, their appropriate migration from the EGL to IGL, and proper apoptosis of GCPs or mature granule cells (Iskusnykh et al., 2021).

Consistently with a reduced number of mature granule cells in the IGL of preterm animals, decreased proliferation was detected in the EGL of preterm human subjects, preterm pigs, and preterm rabbits (Haldipur et al., 2011; Sveinsdóttir et al., 2017; Iskusnykh et al., 2018). Decreased proliferation of GCPs in pigs was associated with reduced expression of cell-cycle promoting cyclins and the *Atoh1* and *Jag1* genes (Iskusnykh et al., 2018). The Atoh1 transcription factor is critical for the expansion of GCPs, and the secreted mitogen jagged canonical notch ligand 1 (Jag1) promotes the proliferation of GCPs in the mouse (Solecki et al., 2001; Flora et al., 2009; Klisch et al., 2011; Višnjar et al., 2022). Interestingly, supplementation of *ex vivo* cerebellar slices from preterm pigs with recombinant Jag1 protein rescued GCP proliferation, further supporting the role of Jag1 as an essential mediator of the preterm cerebellar hypoplasia phenotype (Iskusnykh et al., 2018). Notably, contrary to the results obtained in healthy preterm pigs (Bergström et al., 2016; Iskusnykh et al., 2018), postmortem examination of cerebellar tissue from preterm infants revealed reduced expression of the secreted mitogen Shh in Purkinje cells and reduced Shh signaling in the EGL (Haldipur et al., 2011). Since preterm human infants frequently experience hemorrhage, sepsis/inflammation, and treatment with glucocorticoids, the disruption of their cerebellar Shh signaling may be caused by one or more of these confounding factors rather than directly by preterm birth and the premature exposure to the extrauterine environment *per se*.

In the EGL of both preterm humans and pigs, the differentiation of granule cells appeared to be normal based on immunohistochemical detection of differentiation markers axonal glycoprotein 1 (Tag1), doublecortin (DCX), and β-tubulin (Haldipur et al., 2011; Iskusnykh et al., 2018). Nor was any difference in the number of apoptotic cells in the EGL detected between preterm and control pigs (Iskusnykh et al., 2018). While the migration of granule cells from the EGL to IGL, which is underway in the third trimester of human pregnancy, was not specifically tested in preterm animals, Bergmann glial fibers that guide the radial migration of granule cells were decreased in numbers in both preterm humans and preterm pigs (Haldipur et al., 2011; Iskusnykh et al., 2018). Thus, similar to reduced proliferation of GCPs, altered migration of granule cells from the EGL to IGL might contribute to the cerebellar hypoplasia associated with preterm birth.

Notably, fetuses develop *in utero* in relatively hypoxic conditions and arterial oxygen tension sharply increases upon birth (Scheuer et al., 2015). Analysis of neonatal rodent models of hyperoxia revealed that an increased oxygen concentration reduces proliferation in the EGL. Mechanistically, hyperoxia reduces the expression of cell-cycle promoting Cyclin D2 and the Notch target transcription factor hairy and enhancer of split-1 (*Hes1*) (Scheuer et al., 2015). These observations suggest that at least some adverse effects of preterm birth may be mediated by the early exposure of the immature cerebellum to elevated oxygen levels.

### Purkinje cells

Multiple lines of evidence indicate that preterm birth negatively affects Purkinje cells. While analysis of postmortem cerebellar tissue from preterm infants and preterm pigs showed no difference in the overall density of Purkinje cells, the number of glutamate decarboxylase 67 (GAD67)—positive Purkinje cells was specifically reduced in the posterior cerebellum of preterm guinea pigs of both sexes (Haldipur et al., 2011; Iskusnykh et al., 2018; Shaw et al., 2018). Considering the importance of the GAD67 enzyme for secretion of the neurotransmitter gamma-aminobutyric acid (GABA), these data suggest that dysregulation of GABAergic tone in the cerebellum partially mediates the negative consequences of preterm birth.

In a preterm rabbit model, Purkinje cells exhibited decreased immunostaining for the calcium binding protein Calbindin, suggesting their delayed maturation (Sveinsdóttir et al., 2017). In a non-human primate model, preterm baboons that experienced 2 weeks of neonatal intensive care exhibited reduced growth of Purkinje cell dendrites and electrophysiological abnormalities that affected both synaptic input and output (Barron and Kim, 2020). The authors proposed that the delayed developmental refinements of Purkinje cells result in functional deficits in preterm subjects. Interestingly, genetic depletion of granule cells in the mouse prevented the development of typical Purkinje cell morphology, proper Purkinje cell connectivity and firing activity, and resulted in impaired motor coordination and vocal skills (van der Heijden et al., 2021). Thus, compromised development and functional properties of Purkinje cells in preterm subjects may at least partially result from granule cell developmental defects. Similar to granule cells, some adverse effects of preterm birth on the development of Purkinje cells may be caused by an elevated oxygen concentration in the *ex utero* environment relative to *in utero* conditions, as hyperoxia was reported to inhibit the maturation and dendrite branching of Purkinje cells (Scheuer et al., 2015).

### Other cerebellar neurons

Little is known about the effect of preterm birth on other types of cerebellar neurons. One study revealed no difference in the number of differentiating precursors of molecular layer interneurons (Pax2+ precursors of basket and stellate cells) in the vermis of preterm pigs relative to newborn term controls (Iskusnykh et al., 2018). However, the number of mature basket or stellate cells or their earlier progenitors in the white matter was not investigated in either preterm animal models or postmortem human cerebellar tissue. Golgi, Lugaro, and unipolar brush cells have also not been analyzed in preterm subjects.

### Glial cells

The different glial cell types that comprise the cerebellum are also affected by preterm birth. Particularly, preterm human infants exhibited a reduced density of Bergmann glial fibers in the cerebellar molecular layer (Haldipur et al., 2011). Since Shh is necessary for proper development of Bergmann glia (Chizhikov et al., 2007; Cheng et al., 2018; Lee et al., 2022), the impaired Shh signaling observed in preterm infants (Haldipur et al., 2011) may contribute to their cerebellar Bergmann glial cell pathology. Preterm pigs also exhibited reduced numbers of Bergmann glial fibers, which was associated with a reduced expression of the secreted Notch ligand Jag1 (Iskusnykh et al., 2018). Since differentiation, migration, and survival of Bergmann glia depend on Jag1/Notch signaling (Weller et al., 2006; Komine et al., 2007; Yu et al., 2018), the abnormalities in the Bergmann glial cells in preterm pigs may be mediated by decreased Notch signaling.

Preterm birth also affected the development of oligodendrocytes, which are myelin-forming glial cells residing in the white matter of the cerebellum (Grimaldi et al., 2009; Scheuer et al., 2015; Hashimoto et al., 2016; Shaw et al., 2018). Immature oligodendrocytes are particularly sensitive to the oxidative stress that results from premature exposure to the extrauterine environment and respond with increased apoptosis, reduced proliferation, and delayed differentiation (Nitsos and Rees, 1990; Mallard et al., 2000; Gerstner et al., 2006; Ritter et al., 2013; Scheuer et al., 2015).

## Adverse effects of other factors commonly associated with preterm birth

In addition to directly affecting the cerebellar developmental program as described above, preterm birth is also associated with various complications or other factors that may adversely affect cerebellar development. Some of the most common of these adverse factors are cerebellar hemorrhage, inflammation, and the side effects of medical treatments. Although these factors are not uniquely applicable to preterm subjects and not all preterm infants experience one or more of these factors, preterm babies have a much higher chance of experiencing these factors than term infants. Thus, adverse effects on cerebellar development of hemorrhage, inflammation, and side effects of medical treatments are discussed briefly below.

### Hemorrhage

Cerebellar hemorrhage is a common complication of preterm birth that affects up to 37% of preterm babies (Boswinkel et al., 2019; Gano and Barkovich, 2019; Brossard-Racine and Limperopoulos, 2021). The immature brain vasculature of preterm babies is particularly prone to damage when blood flow fluctuates following birth (Rhee et al., 2018). Bleeding can occur directly in the cerebellar tissue (intraparenchymal hemorrhage) or in adjacent regions (subarachnoid or intraventricular hemorrhages) (Spoto et al., 2021). Bleeding within the brain parenchyma causes injury to the white matter, while massive cerebellar hemorrhage results in more generalized cerebellar atrophy that is associated with severe motor and cognitive dysfunction (Limperopoulos et al., 2007; Volpe, 2009, 2021; Gano and Barkovich, 2019; Bonaventura et al., 2022). Negative effects of hemorrhages are mediated by both the toxicity of blood products, such as hemoglobin, for the developing cerebellar cells and the associated increase in reactive oxygen species (Agyemang et al., 2017; Agyemang et al., 2021; Spoto et al., 2021).

Similar to human patients, cerebellar hemorrhage induced by collagenase injection in the mouse resulted in reduced cerebellar growth and persistent neurobehavioral abnormalities (Yoo et al., 2014). The authors described the predominant effect of hemorrhage on granule cell development, associated with the activation of both injury and neuroprotection-associated endogenous mechanisms. In a preterm rabbit model, intraventricular hemorrhage resulted in a decreased proliferation of GCPs in the EGL, delayed maturation of Purkinje cells, and activation of microglia in the white matter (Agyemang et al., 2017), suggesting that cerebellar bleeding has a prominent effect on multiple cellular populations.

### Infection/inflammation

Preterm subjects have a higher chance of experiencing infection/inflammation while still *in utero* or after delivery. Maternal intrauterine infection with systemic fetal inflammation can trigger preterm delivery. On the other hand, the risk of postnatal neonatal infections is increased in subjects born preterm (Volpe, 2009).

Necrotizing enterocolitis with sepsis correlates with a reduced cerebellar diameter and white matter lesions in humans (Shah et al., 2008; Volpe, 2008).

Systemic inflammation in the mouse results in a region-specific volume reduction of both cerebellar white and grey matter (Klein et al., 2022). These cerebellar volume deficits were associated with enrichment of pro-inflammatory markers in microglia, reduced proliferation of oligodendrocyte progenitors, and reduced levels of myelin basic protein and myelin-associated glycoprotein that affected myelination in the neonatal cerebellum (Cardoso et al., 2015; Klein et al., 2022). A similar pattern of microglia activation in the cerebellum was observed in preterm sheep in which intravenous injection of a low dose of *Escherichia coli* lipopolysaccharide (LPS) led to an increased number of microglia, cerebellar white matter injury, and loss of oligodendrocytes (Baburamani et al., 2014; Dean et al., 2014). Although the most prominent target of inflammation is cerebellar white matter, inflammation may also affect other cerebellar regions. For example, intrauterine exposure of pregnant rabbits to LPS reduced the cell size and density of Purkinje cells, which was associated with compromised cerebellar function as tested by eye-blink conditioning (Zhang et al., 2019).

### Side effects of medical treatments

Because of prematurity, newborn preterm infants typically require more diagnostic and treatment procedures than term babies. Unfortunately, some of these treatments can harm cerebellar development. For example, up to 85–100% of preterm infants receive glucocorticoids to prevent or treat lung, airway, or cardiovascular conditions including bronchopulmonary dysplasia and hypotension (Volpe, 2009; Heine et al., 2011). Postnatal exposure of preterm infants to glucocorticoids is associated with reduced cerebellar growth (Tam, 2018). Chronic treatment of neonatal mice with glucocorticoids inhibited Shh-dependent proliferation of GCPs, which exhibit particularly high expression of glucocorticoid receptors, while acute glucocorticoid treatment caused transient increases in apoptosis (Noguchi et al., 2008; Heine and Rowitch, 2009). Interestingly, activation of the Shh-Smo (smoothened, frizzled class G-protein coupled receptor) signaling pathway in transgenic mice or systemic administration of the small molecule Shh agonist (SAG) prevented the adverse effects of glucocorticoids on cerebellar granule cell development (Heine et al., 2011). Another promising approach entails the use of the glucocorticoid prodrug Ciclesonide (brand name Alvesco), which activates glucocorticoid signaling in the lungs but does not lead to apparent cerebellar growth deficits in rats (Jaumotte et al., 2021).

To reduce pain from diagnostic and therapeutic procedures, preterm infants often receive opioids and oral sucrose, both of which affect cerebellar growth in humans or animal models (McPherson et al., 2015; Zwicker et al., 2016; Tremblay et al., 2017; Spoto et al., 2021). Magnetic resonance imaging revealed reduced cerebellar volume in adult mice which repeatedly received oral sucrose shortly after birth (Tremblay et al., 2017). Since no additional brain assessment was performed, the cellular and molecular mechanisms mediating this phenotype remain unknown. The synthetic opioid fentanyl induces apoptosis of cerebellar granule cells in newborn pigs (Sabir et al., 2018), while exposure of the developing rat cerebellum to the opioid analgesic tramadol causes dysgenesis of Purkinje cells, activation of microglia, and abnormalities of Bergmann glia (Aboulhoda and Hassan, 2018).

## Conclusions and future directions

Preterm birth is a significant risk factor for abnormal cerebellar development. In humans, an extensive proliferation of GCPs, radial migration of granule cells, differentiation of Purkinje cells, and folding of the cerebellar cortex are underway in the third trimester of pregnancy, which likely make human cerebellar development particularly vulnerable to adverse effects of preterm birth and the premature exposure to the extrauterine environment. Studies have begun to elucidate the mechanisms that mediate cerebellar pathology in preterm subjects. Preterm birth has been documented to reduce the numbers of specific neuronal (such as granule cells) and glial (such as Bergmann glia and oligodendrocytes) cells and to affect the maturation of neurons (Purkinje cells) and glia (oligodendrocytes). It is likely that premature exposure to an extrauterine environment, including its higher oxygen concentration relative to *in utero*, affects gene expression in distinct cerebellar cell types, resulting in their altered proliferation, differentiation, and increased apoptosis. As such, we hypothesize that abnormal functioning of premature cerebellum results from both intrinsic deficits in cerebellar neurons and the reduced numbers of specific cerebellar cells that form the cerebellar circuitry. Currently, in the preterm cerebellum, functional abnormalities have been documented only in Purkinje cells, while functional properties of other cerebellar neurons are yet to be investigated.

In addition to the early exposure to the extrauterine environment, preterm human subjects may also experience the adverse effects of hemorrhage, inflammation, and the side effects of medical treatments. The common association of preterm birth with other deleterious influences makes it challenging to identify which molecular pathways are specifically affected by preterm birth and which are affected by the confounding factors that frequently accompany preterm birth. Highly controlled studies utilizing large animal models that recapitulate the developmental trajectory of the human cerebellum are particularly needed to dissect the molecular mechanisms compromised by preterm birth and each of its associated factors. A better understanding of the mechanisms of preterm cerebellar pathology will help to develop therapeutic strategies to treat or prevent neurological deficits in babies that are born preterm.
